# Construction and validation of an educational game on biosafety in the central sterile supply department

**DOI:** 10.1590/0034-7167-2023-0478

**Published:** 2024-12-16

**Authors:** Tatiana Nemoto Piccoli Moraes, Fernanda Moura D’Almeida Miranda, Rafaela Danta de Sousa, Luciana Schleder Gonçalves, Maria de Fatima Mantovani, Jenira Priscila dos Anjos

**Affiliations:** I Universidade Federal do Paraná. Curitiba, Paraná, Brazil

**Keywords:** Sterilization, Education Continuing, Nursing, Educational Technology, Biosafety., Esterilización, Educación Continua, Enfermería, Tecnología Educacional, Bioseguridad.

## Abstract

**Objectives:**

to construct and validate an educational game on biosafety in the Central Sterile Supply Department of a hospital in Curitiba, PR.

**Methods:**

the study was conducted using a quantitative approach, employing applied and technological research with an exploratory design. The process was divided into six stages, from the definition of the theme to the validation and application of the game. The study was carried out from May to August 2022, involving 17 nursing professionals from a Central Sterile Supply Department during day and night shifts, as well as 9 judges.

**Results:**

the study resulted in the construction of a board game named by the authors as “My Health First.”

**Conclusions:**

the research achieved its objective of constructing and validating an educational game. By reflecting on professional practice and correlating the occupational risks present, the professionals were able to list safe actions, identify problems, and seek solutions.

## RESUMEN

**Objetivos:**

construir y validar un juego educativo sobre bioseguridad en la Central de Materiales y Esterilización en un hospital de Curitiba-PR.

**Métodos:**

el trabajo se llevó a cabo mediante un enfoque cuantitativo, utilizando investigación aplicada y tecnológica, con un diseño exploratorio. El proceso se dividió en seis etapas, desde la definición del tema hasta la validación y aplicación del juego. El estudio se realizó de mayo a agosto de 2022, con 17 profesionales de Enfermería de una Central de Materiales y Esterilización en turnos diurnos y nocturnos, además de 9 jueces.

**Resultados:**

el estudio resultó en la construcción de un juego de mesa nombrado por los autores como “Mi Salud en Primer Lugar”.

**Conclusiones:**

la investigación alcanzó el objetivo propuesto de construir y validar un juego educativo. Al reflexionar sobre la práctica profesional y correlacionar los riesgos ocupacionales presentes, los profesionales fueron capaces de enumerar acciones seguras, identificando problemas y buscando soluciones.

## INTRODUCTION

The actions carried out in the Central Sterile Supply Department (CSSD) for the processing of health products (HPs) involve various occupational risks, primarily work-related accidents involving biological material (WRABM)^(1)^. Nursing professionals working in this sector are constantly exposed to materials contaminated with different agents^(2)^.

Biosafety aims to ensure the safety and quality of life for workers^(3)^. These actions, in conjunction with public Worker Health policies, promote the reduction of health workers’ exposure to chemical, physical, biological, and ergonomic agents^(4)^. Biosafety measures are guided by Regulatory Standard (NR-32), which establishes guidelines for protective measures to ensure worker safety and health, such as the use of personal protective equipment (PPE), immunizations, and the proper disposal of sharps^(5)^. It is worth noting that the proper use of PPE provides safety to workers when used correctly^(6)^ and supplied in the necessary quantities by the employer.

The activities carried out in the CSSD are guided and regulated by Collegiate Board Resolution (RDC) No. 15, which includes guidelines on HPs, physical and human resources, and worker safety^(7)^.

Ongoing in-service education is essential for fostering biosafety measures and clarifying the existing occupational risks in the CSSD. Therefore, cleaning and sterilization processes require careful attention to avoid risks to worker health due to the presence of organic matter^(8)^, as well as physical and chemical agents. The effectiveness of ongoing education initiatives in the workplace depends on the participation of the nursing team along with management^(9)^.

The use of educational technologies influences process changes for better health care quality^(10)^. Thus, the use of active methodologies promotes reflection on the issues presented and discussions on strategies for improving professional practice^(6)^.

The use of games as an educational tool enhances clinical reasoning alongside learning, stimulating various skills in participants and requiring active and critical participation^(11)^. Games also help improve concentration and develop various skills. The challenge of achieving the game’s objective creates a motivating atmosphere among participants^(11)^.

## OBJECTIVES

To construct and validate an educational game on biosafety in a Central Sterile Supply Department (CSSD) of a hospital in the Metropolitan Region of Curitiba, Paraná (PR).

## METHODS

### Ethical Aspects

The study, derived from a Master’s thesis, was conducted in accordance with national and international ethical guidelines. It was approved by the Research Ethics Committee (REC) of the Health Sciences Sector at UFPR, with the approval attached to this submission, and it followed Resolution No. 466 of 2012 from the National Health Council^(7)^.

### Design, Study Location, and Period

This is an applied, technological research study with an exploratory design and a mixed-methods approach, incorporating both quantitative and qualitative methods. The methodological framework for creating this educational game was based on a study^(12)^ comprising six stages: 1 - defining the theme, objectives, and purposes of the educational game; 2 - conducting a literature review on games related to the theme; 3 - designing the educational game; 4 - constructing the educational game; 5 - validating the educational material; and 6 - applying the game. The construction of the game was guided by the Consolidated Criteria for Reporting Qualitative Research (COREQ), a tool that suggests standards for conducting qualitative studies^(13)^.

In the development of the educational game, the following characteristics were considered: Content, representing the reality of the proposed theme; Language, easily understood by the player; Organization, which captures the player’s attention (layout and illustration); and, finally, Learning, which is the focus of this educational game^(12)^. The construction and validation of the educational game took place from May 2022 to August 2022, in a CSSD of a municipal hospital in the metropolitan region of Curitiba, Paraná, Brazil.

### Population

For the content validation stage, the judges were selected through convenience sampling. The invitation to participate was sent via email and included a form on the Google Forms^®^ platform. The exclusion criterion was the failure to return the validation instrument within the stipulated period for the respective round.

For the game construction stages, participants were invited through a notice posted on the cafeteria bulletin board. The inclusion criteria for participants were: nursing professionals who performed activities in the CSSD. Workers who were on vacation or medical leave were excluded from this research.

The participants were divided into two groups. Group A participated in the development of the educational game content, while Group B conducted the evaluation of the educational game’s appearance.

Group A consisted of eight nursing professionals, and Group B consisted of nine nursing professionals.

Informed consent was obtained from all individuals involved in the study: from the group participants through a printed form, and from the judges through an online form.

### Study protocol

#### STEP 1: Definition of the theme, objectives, and purposes of the game

To assess the nursing professionals’ knowledge, a questionnaire was made available via Google Forms^®^. This questionnaire included socio-occupational information, characterization of WRABM, and general biosafety knowledge. The responses were grouped according to a Likert scale, with five options: completely unaware, partially unaware, indifferent, partially aware, and fully aware.

The theme of biosafety, selected for the game, reflected the daily work activities carried out in the CSSD.

#### STEP 2: Literature review on the application of games in continuing education

A search was conducted for existing research related to educational games in healthcare, specifically targeting healthcare professionals. The search for studies took place between March and April 2021. A total of 1,247 studies were found, with 48 in the BVS, 695 in the Web of Science, and 504 in PubMed. After analysis, 13 articles relevant to the theme were selected.

#### STEP 3: Design of the educational game

The construction of the game prototype began after identifying knowledge gaps highlighted by the questionnaire responses, in conjunction with the scientific literature reviewed.

The game was composed of a board with a path to be followed and 26 cards with questions related to biosafety and occupational risks specific to the CSSD. In addition to the players, a mediator was needed to guide and oversee the educational action of the game. To facilitate this, a booklet with guidelines and expected answers was created for the mediator to use during the game.

#### STEP 4: Construction of the educational game

This stage was organized into three workshops with the participation of Group A (Chart 1). The workshops followed scripts organized by the researcher, with discussions based on the questionnaire results and the prototype game cards. The workshops were held in the workplace, each lasting approximately one hour. The strategy used for the script was the problematization methodology, employing the Maguerez Arc tool. Created by Bordenave and Pereira^(14)^, the Maguerez Arc allows problems from reality to be addressed systematically. In the final workshop, the prototype test was conducted.

**Chart 1:** Summary of the objectives proposed in the workshops for the construction of the educational game related to the Maguerez Arc.

1st Workshop	1st Phase of AM: Observation of Reality 2nd Phase of AM: Key Points
2nd Workshop	3rd Phase of AM: Theorization
3rd Workshop	4th Phase of AM: Hypotheses of Solution

**Quadro 2:** Resultado da avaliação dos juízes sobre o conjunto carta+pergunta+orientação

Set	CVI (n=8)	Action
1	0.88	Retained
2	0.88	Retained
3	0.75	Discarded
4	1.00	Retained
5	0.88	Retained
6	1.00	Retained
7	1.00	Retained
8	1.00	Retained
9	1.00	Retained
10	1.00	Retained
11	0.88	Retained
12	0.63	Discarded
13	0.88	Retained
14	0.88	Retained
15	0.88	Retained
16	0.88	Retained
17	1.00	Retained
18	0.63	Discarded
19	0.63	Discarded
20	0.88	Retained
21	1.00	Retained
22	0.63	Discarded
23	1.00	Retained
24	0.63	Discarded
25	1.00	Retained
26	0.88	Retained

The participation of groups representing the target audience for which the educational technology is intended is essential to achieving the proposed objectives^(15)^. A structured instrument, workshop recordings, and field diary notes were used to make the necessary adjustments to the game.

#### STEP 5: Validation

Judges were invited to evaluate the content of the following elements: explanatory text, questions, and mediator guidelines. The Delphi technique was employed, in which analysis rounds are conducted as many times as necessary to reach a consensus. Validation is achieved through consensus among experts using an instrument. An item is considered valid when the responses show consensus according to the Content Validity Index (CVI)^(16)^.

The content validation questionnaire was organized according to a Likert scale. Each judge expressed their evaluation on a scale of 1 to 4, with 1 - Fully adequate, 2 - Adequate, 3 - Partially adequate, and 4 - Inadequate, to calculate the Content Validity Index (CVI). The CVI is calculated by summing the agreement of items marked as “1” and “2” and dividing it by the total number of responses. The acceptable agreement index among the judges must be at least 0.80, and preferably greater than 0.90^(17)^.

#### STEP 6: Application to the Target Audience

The game was presented to the group of participants represented by Group B, who conducted the appearance evaluation using an instrument adapted by the researcher. This evaluation allows the target audience to verify whether the content represents the reality in which it will be used^(18)^.

#### Results analysis

The data collected through a questionnaire made available on Google Forms^®^ was analyzed using Microsoft Excel software. Absolute and relative frequency analyses were performed.

For the validation of the results obtained by the judges, the Content Validity Index (CVI) was used. The CVI is a way to quantify the agreement among judges on specific domains after a qualitative approach^(18)^. The agreement rate is calculated by determining the percentage for each evaluated domain. In this study, the following elements were evaluated: the question, the mediator’s text, and the answer. A result with a consensus of 80% or more was considered valid.

The game was validated in the first evaluation round, with some adjustments suggested by the expert judges.

The qualitative approach encompassed the construction of the game using the content provided in the four workshops. The participants’ speeches were transcribed, processed, and analyzed using the IRaMuTeQ software, employing similarity analysis and word clouds. Similarity analysis is based on grouping words within the same context^(19)^.

## RESULTS

The study resulted in the creation of a board game named by the authors as “My Health First”.

### Population Characterization

The information obtained from the questionnaire responses was used to design the game prototype and served as a basis for discussing the real-life challenges during the workshops. A total of 21 questionnaires were completed, representing 60% of the nursing professionals working in the CSSD.

The majority of respondents were female (90.5%), with more than 11 years of professional experience (95.2%) and more than 6 years working in the CSSD sector (42.9%). Most (43%) had experienced WRABM once, while 24% had experienced it two or more times, totaling 66%. The causes identified included: third-party errors (48%), technical failures (14%), and lack of PPE (10%).

The last part of the questionnaire highlighted the lack of knowledge among nursing professionals regarding their work environment, the existing occupational risks, and the necessary preventive actions. Only 14% fully understood the definition of biosafety, and 76% were aware of the occupational risks in the service. It was also noted that there was a lack of knowledge regarding the regulations pertinent to NR 32 (48%) and RDC 15 (74%).

### The Workshops

During the workshops, participants analyzed the game prototype and reported that the content of the cards was consistent with the reality experienced by nursing professionals in the CSSD.

**Figure 1:**
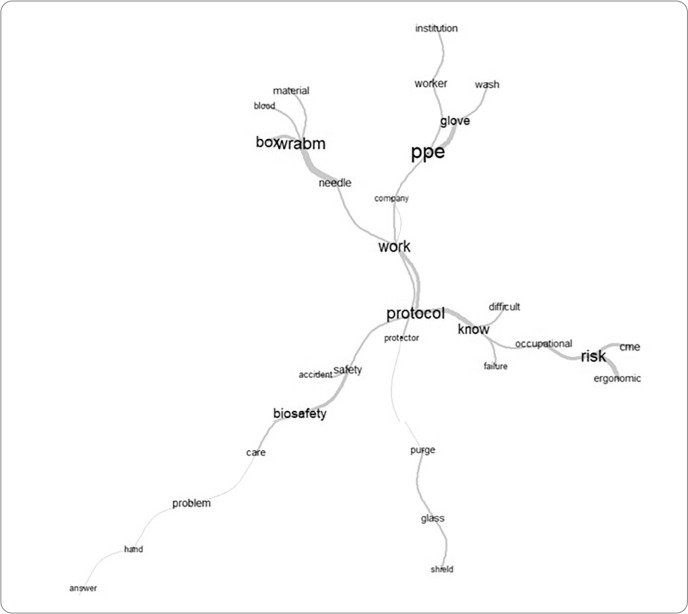
Similarity analysis of the discourse from the 2nd workshop

The transcription and preparation of the material recorded in the first workshop were processed using the IRaMuTeQ software, resulting in a similarity analysis. The word “protocol” stood out centrally in the discourse, giving rise to other branches, such as the words: “PPE”, “risk”, “WRABM”, and “biosafety”, which supported the themes proposed in the game prototype (Figure 1).

The workshops provided important data for improving the game cards, showing that the issues discussed were cohesive with the scientific literature presented on the cards.

### Validation

The expert judges who participated in the validation of the game were mostly female (n=6), and the majority were between 41 and 50 years old (n=5). All had a degree in Nursing, with seven holding a Ph.D. and one a specialist degree. All were affiliated with public institutions, with one working in the CSSD (n=1) and six in teaching, specializing in occupational health (n=6).

The content validation of the cards showed a CVI percentage of ≥ 0.80 agreement, ranging from 0.88 to 1.00 in most sets (Chart 2). Among these, 10 sets achieved a CVI of 1.00, and 10 sets achieved a CVI of 0.88. The six cards that did not reach a CVI of ≥ 0.80 were discarded, and no further rounds among the judges were necessary. With these results, all the content was validated in a single round, resulting in 20 validated sets.

### Prototype Testing

The board game “My Health First” was tested twice. The first test was the pilot test, during which problems were identified with some of the cards and the game’s strategy. The modification applied after the pilot test was a change to the bonus activity: previously, three examples were required, and this was modified to one practical example. According to Pires, Gottems, and Fonseca^(20)^, the prototype should be tested and evaluated before its final application to ensure that it meets the proposed objective; if necessary, improvements can be made.

The second test was conducted by Group B, who played the game and performed the appearance validation using an instrument adapted by the researcher. The instrument was a Likert-type scale, consisting of 18 questions divided into five sections: objective, organization, writing style, appearance, and motivation.

As a result, the game received 100% approval in the sections of objectives, organization, appearance, and motivation, achieving the goal of having an educational characteristic in the work process. The importance of the game being easy to understand and assimilate the proposed content was highlighted, as reported by the participants:

*Very good, it makes us think about the ‘whys’ and the purposes of each procedure.* (A6)

[...] *a moment of relaxation with the team* [...]. (A4)

## DISCUSSION

This research developed and validated an educational game on biosafety for nursing professionals in the CSSD, titled “My Health First.” The discussions held during the workshops provided a critical perception of reality, particularly concerning workplace safety and the importance of using PPE. The contributions from the nursing professionals in the groups brought the reality closer to the concepts fostered by each professional’s knowledge. Creating spaces for discussion broadens the perspective on the workplace and leads to improvements in service quality^(21)^.

A study conducted in Goiás by Carvalho and colleagues^(22)^ on the predictors of WRABM recurrence interviewed 73 healthcare professionals who received care at the Specialized Care Service (SCS) after experiencing WRABM. It was found that 32.9% of the respondents had previously experienced WRABM, and when analyzed together with the presence of ongoing education in the workplace, a significant difference was observed between healthcare professionals who had recurrent incidents and those who did not participate in ongoing education. Similar to the present study, 43% of the participants had experienced WRABM once, and 24% had experienced it two or more times.

Educational activities increase workers’ knowledge and skills, improving the quality of the tasks performed. In this way, ongoing education encourages the development of good professional practices, refining everyday situations in conjunction with scientific evidence^(23)^.

Opportunities for ongoing education must be provided by managers so that these training sessions are offered during working hours. However, maintaining and continuing ongoing education is a challenge for institutions^(24)^. The PNEPS (National Policy on Permanent Health Education) ordinance provides and describes other determinations to stimulate quality training, highlighting its social value, a guideline advocated by the Unified Health System (SUS)^(25)^.

In this context, the use of educational games as an educational strategy has proven effective in knowledge absorption when compared to other traditional teaching methods. A study by Bellan and colleagues^(26)^, conducted with 30 nursing professionals in the interior of São Paulo, used a card game to assess knowledge retention before and after the application of the game, resulting in a 50% improvement in the post-test. The use of games enhances the dynamics and achievement of the objectives proposed in educational activities^(27)^.

Corroborating this research, the study by Brull and colleagues^(28)^ compared three methodologies regarding the retention of knowledge among participants in the post-test. Traditional lectures, online classes, and an interactive game were compared. All groups, despite receiving the same content, showed differences in post-test results. The group that used the game had a higher number of correct answers compared to the other two groups.

The use of educational materials requires methodological care to meet the intended objectives. The construction of the board game, using the method proposed by Andrade and colleagues^(12)^, presents the essential steps for creating a pedagogical product. In this way, playful activities foster professional skills such as critical analysis, social relationships, creativity, imagination, and motivation^(27)^.

The validation of educational technology is necessary for the legitimacy of the product. All items must be representative and clear to be adequately used for their intended function. The contributions incorporated allow for the scientific conception of the product, supporting its educational reach^(29)^. The construction and content validation of the game were deemed appropriate, with consensus among the judges on the topics presented, requiring only one round among the specialists. The content validation represented the integration of the researcher’s practical activities with the theoretical scientific framework. Twenty cards reached the recommended consensus, while six were excluded for not achieving the necessary CVI. The judges expressed their opinions on most of the cards, and the suggested adjustments were made.

The evaluation conducted by the target audience of the product is important to identify the need for adjustments, ensuring that the material is practical and accessible^(30)^. The focus is not on questioning the content but rather on expressing opinions about the objectives, organization, writing style, appearance, and motivation^(15)^. The feedback provided by the participants in Group B marked the conclusion of the game development process, contributing to a positive response to the educational initiative.

### Study limitations

The game was constructed based on local reality, addressing the needs presented by the participants.

### Contributions to the Field of Nursing, Health, or Public Policy

This product can be utilized by various health services across the country, with the aim of enhancing nursing professionals’ knowledge of biosafety measures in the CSSD. It is also suggested that future studies investigate and compare learning outcomes using educational games.

## CONCLUSIONs

The research achieved its objective of constructing and validating an educational game. The board game “My Health First” has demonstrated its effectiveness in helping nursing professionals recognize vulnerabilities related to biosafety in the Central Sterile Supply Department (CSSD) work environment, encouraging them to adopt safety measures to prevent illness and work-related accidents.

The group discussions in this research contributed to necessary observations for the prototype, bringing it closer to the reality experienced in professional practice. The participants’ concerns were reflected in the game cards, confirming the content selected by the researcher during card development. By reflecting on professional practice and correlating the existing occupational risks, the professionals were able to promote safe actions, identify problems, and seek solutions.

The active participation of the nursing professionals involved in the game development facilitated the understanding of the real needs of professional practice. Often, educational activities are developed theoretically without addressing local reality. Training activities, whether through continuing or permanent education, present a challenge for managers. The participation of workers in these training sessions is hindered by the routines imposed by the service, which do not allow space for permanent education in the workplace, leading to a lack of motivation among health professionals to participate.
