# Supplementary material for: Construction and validation of an educational game on biosafety in the central sterile supply department
Source: Rev Bras Enferm. 2024 Dec 16;77(6):e20230478. doi: 10.1590/0034-7167-2023-0478 (PMC11654519; doi:10.1590/0034-7167-2023-0478)
Supplement: Translation

# Translation: Construção e validação de um jogo educativo sobre biossegurança na central de materiais e esterilização

**DOI:** 10.1590/0034-7167-2023-0478pt

**Authors:** Tatiana Nemoto Piccoli Moraes, Fernanda Moura D’Almeida Miranda, Rafaela Danta de Sousa, Luciana Schleder Gonçalves, Maria de Fatima Mantovani, Jenira Priscila dos Anjos

**Affiliations:** I Universidade Federal do Paraná. Curitiba, Paraná, Brasil

**Keywords:** Esterilização, Educação Continuada, Enfermagem, Tecnologia Educacional, Biossegurança.

## Abstract

**Objetivos:**

construir e validar um jogo educativo sobre biossegurança na Central de Materiais e Esterilização em um hospital de Curitiba-PR.

**Métodos:**

o trabalho foi conduzido por meio de uma abordagem quantitativa, utilizando pesquisa aplicada e tecnológica, com um desenho exploratório. O processo foi dividido em seis etapas, desde a definição do tema até a validação e aplicação do jogo. O estudo foi realizado de maio a agosto de 2022, com 17 profissionais de Enfermagem de uma Central de Materiais e Esterilização nos turnos diurnos e noturnos, além de 9 juízes.

**Resultados:**

o estudo resultou na construção de um jogo de tabuleiro nomeado pelos autores como “Minha Saúde em Primeiro Lugar”.

**Conclusões:**

a pesquisa atingiu o objetivo proposto de construir e validar um jogo educativo. Ao refletirem sobre a prática profissional e correlacionarem os riscos ocupacionais presentes, os profissionais foram capazes de elencar ações seguras, identificando problemas e buscando soluções.

## INTRODUÇÃO

As ações desenvolvidas na Central de Materiais e Esterilização (CME) para o processamento de produtos para a saúde (PPS) acarretam diferentes riscos laborais, principalmente os acidentes de trabalho com material biológico (ATMB)^(1)^. Os trabalhadores de enfermagem que desempenham suas atividades nesse setor estão constantemente expostos a materiais contaminados com diferentes agentes^(2)^.

A biossegurança visa garantir a segurança e a qualidade de vida do trabalhador^(3)^. Essas ações, em conjunto com as políticas públicas de Saúde do Trabalhador, promovem a redução do contato dos trabalhadores de saúde com agentes químicos, físicos, biológicos e ergonômicos^(4)^. As ações de biossegurança são orientadas pela Norma Regulamentadora (NR-32), que estabelece diretrizes para medidas de proteção à segurança e à saúde do trabalhador, como o uso de equipamentos de proteção individual (EPIs), imunizações e o descarte correto de perfurocortantes^(5)^. Destaca-se que o uso adequado de EPIs proporciona segurança ao trabalhador quando são utilizados corretamente^(6)^ e fornecidos na quantidade necessária pelo empregador.

As atividades desenvolvidas na CME são orientadas e regulamentadas pela Resolução da Diretoria Colegiada (RDC) nº 15, que inclui orientações sobre o PPS, recursos físicos e humanos, e segurança do trabalhador^(7)^.

A educação permanente em serviço é essencial para fomentar as medidas de biossegurança e explicitar os riscos ocupacionais existentes na CME. Dessa forma, os processos de limpeza e esterilização exigem cuidados e atenção redobrados, a fim de evitar riscos à saúde do trabalhador devido à presença de matéria orgânica^(8)^, agentes físicos e químicos. As ações de educação permanente desenvolvidas no ambiente de trabalho dependem da participação da equipe de enfermagem em conjunto com os gestores^(9)^ para que sejam efetivas.

A utilização de tecnologias educacionais influencia as mudanças de processos para uma melhor qualidade de atenção à saúde^(10)^. Dessa forma, a utilização de metodologias ativas promove a reflexão sobre os problemas apresentados e discute estratégias de melhoria para a prática profissional^(6)^.

O uso de jogos como ferramenta educativa reforça o raciocínio clínico em conjunto com a aprendizagem, estimulando diferentes habilidades dos interlocutores, exigindo uma participação ativa e crítica^(11)^. Os jogos também auxiliam na concentração e no desenvolvimento de diversas habilidades. O desafio de atingir o objetivo do jogo cria uma atmosfera motivadora entre os participantes^(11)^.

## OBJETIVOS

Construir e validar um jogo educativo sobre biossegurança em uma Central de Material e Esterilização de um hospital da Região Metropolitana de Curitiba, Paraná (PR).

## MÉTODO

### Aspectos éticos

O estudo, originado de uma dissertação de Mestrado, foi conduzido de acordo com as diretrizes de ética nacionais e internacionais, aprovado pelo Comitê de Ética em Pesquisa (CEP) do Setor de Ciências da Saúde da UFPR, cujo parecer está anexado à presente submissão, e seguiu a Resolução nº 466 de 2012, do Conselho Nacional de Saúde^(7)^.

### Desenho, local do estudo e período

Trata-se de uma pesquisa aplicada, tecnológica, de desenho exploratório e abordagem quantitativa e qualitativa. O referencial metodológico para a criação deste jogo educativo foi baseado em um estudo^(12)^ que contempla seis etapas: 1 - definição do tema, objetivos e finalidades do jogo educativo; 2 - pesquisa na literatura sobre jogos relacionados ao tema; 3 - elaboração do jogo educativo; 4 - construção do jogo educativo; 5 - validação do material educativo; e 6 - aplicação do jogo. As recomendações dos critérios consolidados para relatar pesquisas qualitativas (COREQ), uma ferramenta que sugere padronizações para a realização de estudos qualitativos, nortearam a construção do jogo^(13)^.

Para a elaboração do jogo educativo, foram consideradas as seguintes características: Conteúdo, que representa a realidade do tema proposto; Linguagem, de fácil compreensão pelo jogador; Organização, que chama a atenção do jogador (layout e ilustração); e, por fim, Aprendizagem, que é o foco deste jogo educativo^(12)^.

A construção e validação do jogo educativo ocorreram no período de maio de 2022 a agosto de 2022, em uma Central de Material e Esterilização de um hospital municipal da região metropolitana de Curitiba, no estado do Paraná, Brasil.

### População

Para a etapa de validação de conteúdo, os juízes foram selecionados por amostra de conveniência. O convite à participação foi enviado via e-mail e incluiu um formulário na plataforma *Google Forms* ^®^. Como critério de exclusão, foi considerada a não devolução do instrumento de validação no período estipulado para a respectiva rodada.

Para as etapas de construção do jogo, os participantes foram convidados por meio de um convite fixado no painel de avisos do refeitório. Os critérios de inclusão dos participantes foram: profissionais de enfermagem que desenvolviam atividades na CME. Foram excluídos desta pesquisa trabalhadores que estavam em período de férias ou licença médica.

Os participantes foram divididos em dois grupos. O Grupo A participou do desenvolvimento do conteúdo do jogo educativo, enquanto o Grupo B realizou a avaliação da aparência do jogo educativo.

O Grupo A foi composto por oito profissionais de enfermagem, e o Grupo B, por nove profissionais de enfermagem.

O Consentimento Livre e Esclarecido foi obtido de todos os indivíduos envolvidos no estudo: dos participantes dos grupos, por meio de um formulário impresso, e dos juízes, por um formulário online.

### Protocolo de estudo

#### ETAPA 1: Definição do tema, objetivos e finalidades do jogo

Para caracterizar os conhecimentos dos profissionais de enfermagem, foi disponibilizado um questionário pelo *Google Forms* ^®^. Este era constituído de informações sócio-ocupacionais, caracterização dos ATMB e conhecimentos gerais de biossegurança. As respostas foram agrupadas conforme a escala de Likert, na qual havia cinco opções: desconheço totalmente, desconheço parcialmente, indiferente, conheço parcialmente e conheço totalmente. O tema biossegurança, selecionado para o jogo, refletiu o cotidiano das atividades laborais realizadas na CME.

#### ETAPA 2: Pesquisa na literatura sobre a aplicação de jogos na educação permanente

Realizou-se uma busca das pesquisas existentes relacionadas aos jogos educativos na saúde voltados aos profissionais da saúde. A busca dos estudos ocorreu entre os meses de março e abril de 2021. Foram encontrados 1.247 estudos, sendo 48 na BVS, 695 na *Web of Science* e 504 na *PubMed*. Após análises, foram selecionados 13 artigos pertinentes ao tema.

#### ETAPA 3: Elaboração do jogo educativo

A construção do protótipo do jogo teve sua concepção após a identificação das lacunas de conhecimento apresentadas nas respostas do questionário, em conjunto com a literatura científica pesquisada.

O jogo foi composto por um tabuleiro com um caminho a ser percorrido e 26 cartas com perguntas relacionadas a biossegurança e riscos ocupacionais referentes à CME. Além dos jogadores, era necessário um mediador para acompanhar e orientar a ação educativa do jogo. Para esse fim, foi criado um folheto com orientações e respostas esperadas para que o mediador utilize durante o jogo.

#### ETAPA 4: Construção do jogo educativo

Esta etapa foi organizada em três oficinas com a participação do Grupo A (Quadro 1). As oficinas seguiam roteiros organizados pela pesquisadora, com discussões baseadas nos resultados do questionário e nas cartas do protótipo do jogo. As oficinas foram realizadas no local de trabalho, com cerca de uma hora de duração. A estratégia utilizada para o roteiro foi a metodologia da problematização com a ferramenta do Arco de Maguerez. Criado por Bordenave e Pereira^(14)^, o Arco de Maguerez permite trabalhar os problemas da realidade de forma esquemática. Na última oficina, foi realizado o teste do protótipo.

**Quadro 1:** Síntese dos objetivos propostos nas oficinas para a construção do jogo educativo relacionados ao Arco de Maguerez

1ª Oficina	1ª Fase do AM: Observação da Realidade 2ª Fase do AM: Pontos Chaves
2ª Oficina	3ª Fase do AM: Teorização
3ª Oficina	4ª Fase do AM: Hipóteses de Solução

A participação dos grupos que representam o público-alvo ao qual se destina a produção da tecnologia educacional é essencial para que se alcancem os objetivos propostos^(15)^. Optou-se pela utilização de um instrumento estruturado, gravações das oficinas e anotações em diário de campo para que fosse possível realizar as adequações necessárias no jogo.

#### ETAPA 5: Validação

Os juízes foram convidados a avaliar o conteúdo do conjunto: texto explicativo, perguntas e orientações do mediador. Utilizou-se a técnica Delphi, na qual são realizadas rodadas de análise quantas forem necessárias para atingir o consenso. A validação consiste no consenso entre os especialistas por meio de um instrumento. O item é considerado válido quando as respostas apresentam consenso conforme o Índice de Validação de Conteúdo (IVC)^(16)^.

O questionário para validação de conteúdo foi organizado conforme a escala de Likert. Cada juiz expressou sua avaliação em graus de 1 a 4, sendo 1 - Totalmente adequado, 2 - Adequado, 3 - Parcialmente adequado e 4 - Inadequado, de modo a obter o Índice de Validade de Conteúdo (IVC). O IVC é calculado por meio do somatório de concordância dos itens marcados como “1” e “2”, dividido pelo total de respostas. No total de respostas, o índice de concordância aceitável entre os juízes deve ser de no mínimo 0,80 e, preferencialmente, maior que 0,90^(17)^.

#### ETAPA 6: Aplicação ao público-alvo

O jogo foi apresentado ao grupo de participantes representado pelo Grupo B, que realizou a avaliação de aparência por meio de um instrumento adaptado pela pesquisadora. Essa avaliação permite ao público-alvo verificar se o conteúdo representa a realidade na qual será utilizado^(18)^.

#### Análise de resultados

O resultado da coleta de dados realizada por meio de questionário disponibilizado no *Google Forms* ^®^ foi analisado utilizando o *software Microsoft Excel*. Foram realizadas análises de frequência absoluta e relativa.

Para a validação dos resultados obtidos pelos juízes, foi utilizado o Índice de Validade de Conteúdo (IVC). O IVC representa uma forma de quantificar a concordância entre os juízes sobre determinados domínios, após uma abordagem qualitativa^(18)^. A taxa de concordância é obtida pelo cálculo da porcentagem em cada domínio avaliado. No estudo, foi avaliado o conjunto: a pergunta, o texto do mediador e a resposta. Considerou-se válido um resultado com concordância maior ou igual a 80%.

O jogo foi validado na primeira rodada de avaliação, com algumas adequações apontadas pelos juízes especialistas.

A abordagem qualitativa contemplou a construção do jogo por meio dos conteúdos fornecidos nas quatro oficinas. A transcrição dos discursos dos participantes foi processada e analisada pelo *software* IRaMuTeQ, utilizando a análise de similitude e a nuvem de palavras. A análise de similitude é baseada no agrupamento de palavras com o mesmo contexto^(19)^.

## RESULTADOS

O estudo resultou na construção de um jogo de tabuleiro, nomeado pelos autores como “Minha Saúde em Primeiro Lugar”.

### Caracterização da população

As informações adquiridas por meio das respostas aos questionários foram utilizadas para a concepção do protótipo do jogo e serviram de base para a problematização da realidade durante as oficinas. Foram respondidos 21 questionários, representando 60% dos profissionais de enfermagem que desenvolviam atividades na CME.

A maioria dos respondentes era do sexo feminino (90,5%), com mais de 11 anos de profissão (95,2%) e mais de 6 anos atuando no setor da CME (42,9%). A maioria (43%) já havia sofrido ATMB uma vez, enquanto 24% sofreram duas ou mais vezes, totalizando 66%. As causas apontadas foram: falhas de terceiros (48%), falha técnica (14%) e falta de EPI (10%).

A última parte do questionário evidenciou a falta de conhecimento dos profissionais de enfermagem em relação ao seu ambiente de trabalho, aos riscos ocupacionais existentes e às ações de prevenção necessárias. Apenas 14% conheciam totalmente a definição de biossegurança, e 76% conheciam os riscos ocupacionais do serviço. Verificou-se também o desconhecimento das legislações pertinentes à NR 32 (48%) e à RDC 15 (74%).

### As oficinas

Durante as oficinas, os participantes analisaram o protótipo do jogo e relataram que o conteúdo das cartas estava adequado à realidade vivenciada pelos profissionais de enfermagem na CME.

A transcrição e preparação do material gravado na primeira oficina foram processadas pelo *software* IRaMuTeQ, resultando na análise de similitude. A palavra “protocolo” destacou-se centralmente no discurso, originando outras ramificações, como as palavras: “EPI”, “risco”, “ATMB” e “biossegurança”, corroborando os temas propostos presentes no protótipo do jogo (Figura 1).

**Figura 1:** análise de similitude do discurso da 2ª oficina

As oficinas resultaram em dados importantes para o aprimoramento das cartas do jogo, evidenciando que as questões discutidas estavam coesas com a literatura científica presente nas cartas.

### Validação

Os juízes especialistas que participaram da validação do jogo eram, em sua maioria, do sexo feminino (n=6), e a maioria tinha entre 41 e 50 anos (n=5). Todos possuíam graduação em Enfermagem, sendo sete doutores e um especialista. Todos tinham vínculo com instituição pública, sendo um com atuação assistencial no setor da CME (n=1) e seis com atuação na docência, na área de saúde do trabalhador (n=6).

A validação do conteúdo das cartas apresentou um percentual de IVC ≥ 0,80 de concordância, variando de 0,88 a 1,00 na maioria dos conjuntos (Quadro 2). Dentre esses, 10 conjuntos obtiveram IVC = 1,00 e 10 obtiveram IVC = 0,88. Optou-se por descartar as seis cartas que não atingiram o IVC ≥ 0,80, não sendo necessárias mais rodadas entre os juízes. Com esses resultados, todos os conteúdos foram validados em uma única rodada, resultando em 20 conjuntos validados.

**Quadro 2:** Resultado da avaliação dos juízes sobre o conjunto carta+pergunta+orientação

Conjunto	IVC (n=8)	Ação
1	0,88	Mantido
2	0,88	Mantido
3	0,75	Descartado
4	1,00	Mantido
5	0,88	Mantido
6	1,00	Mantido
7	1,00	Mantido
8	1,00	Mantido
9	1,00	Mantido
10	1,00	Mantido
11	0,88	Mantido
12	0,63	Descartado
13	0,88	Mantido
14	0,88	Mantido
15	0,88	Mantido
16	0,88	Mantido
17	1,00	Mantido
18	0,63	Descartado
19	0,63	Descartado
20	0,88	Mantido
21	1,00	Mantido
22	0,63	Descartado
23	1,00	Mantido
24	0,63	Descartado
25	1,00	Mantido
26	0,88	Mantido

### Teste do protótipo

O jogo de tabuleiro “Minha Saúde em Primeiro Lugar” foi testado duas vezes. A primeira vez foi o teste piloto, no qual foram identificados problemas com algumas cartas e com a estratégia de condução do jogo. A modificação aplicada após o teste piloto foi a alteração da atividade bônus: anteriormente, eram necessários três exemplos, e isso foi modificado para um exemplo prático. Segundo Pires, Gottems e Fonseca^(20)^, o protótipo deve ser testado e avaliado antes da aplicação final para certificar-se de que ele atende ao objetivo proposto; se necessário, aperfeiçoamentos podem ser realizados.

O segundo teste foi realizado pelo Grupo B, que jogou a partida do jogo e realizou a validação de aparência utilizando um instrumento adaptado pela pesquisadora. O instrumento era do tipo Likert, composto por 18 perguntas divididas em cinco seções: objetivo, organização, estilo da escrita, aparência e motivação.

Como resultado, obteve-se uma aprovação de 100% nas seções de objetivos, organização, aparência e motivação, atingindo a finalidade de possuir uma característica educadora no processo de trabalho. Ressalta-se a importância de o jogo ser de fácil compreensão e assimilação dos conteúdos propostos, como relatado pelos participantes:

*Muito bom, faz pensarmos nos “porquês e quais as finalidades de cada procedimento.* (A6)

[...] *momento de descontração com a equipe* [...]. (A4)

## DISCUSSÃO

Esta pesquisa construiu e validou o jogo educativo sobre biossegurança para os profissionais de enfermagem da CME, denominado “Minha Saúde em Primeiro Lugar”. As discussões desenvolvidas nas oficinas trouxeram uma percepção crítica da realidade, principalmente sobre a preocupação com a segurança no trabalho e a importância do uso de EPIs. A contribuição dos profissionais de enfermagem nos grupos aproximou a realidade das concepções fomentadas pelos conhecimentos de cada profissional. Abrir espaços de discussão expande o olhar sobre o local de trabalho e traz melhorias na qualidade do serviço^(21)^.

Um estudo desenvolvido em Goiás, por Carvalho e colaboradores^(22)^, sobre os preditores da reincidência em ATMB, entrevistou 73 profissionais da saúde que tiveram atendimentos no Serviço de Assistência Especializada (SAE) após um ATMB. Foi constatado que 32,9% dos entrevistados já haviam sofrido um ATMB anteriormente e, analisando em conjunto com a presença de educação permanente em serviço, observou-se uma diferença significativa entre os profissionais da saúde reincidentes que não participaram de educação permanente. Semelhante ao presente estudo, 43% dos participantes já haviam sofrido ATMB uma vez e 24% duas ou mais vezes.

As ações educativas aumentam os conhecimentos e as habilidades dos trabalhadores, proporcionando melhoria na qualidade das atividades desempenhadas. Dessa forma, a educação permanente incentiva o desenvolvimento de boas práticas profissionais, aperfeiçoando situações do cotidiano em conjunto com evidências científicas^(23)^.

As oportunidades de educação permanente devem ser proporcionadas pelos gestores, para que essas capacitações sejam oferecidas durante a jornada de trabalho. No entanto, a manutenção e continuidade da educação permanente é um desafio para as instituições^(24)^. A Portaria da PNEPS (Política Nacional de Educação Permanente em Saúde) prevê e descreve outras determinações para o estímulo a uma formação de qualidade, destacando seu valor social, uma diretriz defendida pelo Sistema Único de Saúde (SUS)^(25)^.

Nesse contexto, a utilização de jogos educativos como estratégia educacional mostra-se efetiva na absorção do conhecimento quando comparada a outros métodos tradicionais de ensino. Um estudo de Bellan e colaboradores^(26)^, realizado com 30 profissionais de enfermagem no interior de São Paulo, utilizou um jogo de cartas para avaliar a retenção do conhecimento antes e depois da aplicação do jogo, resultando em uma melhora de 50% no pós-teste. A utilização de jogos favorece a dinâmica e o desenvolvimento dos objetivos propostos nas atividades educativas^(27)^.

Corroborando com esta pesquisa, o estudo de Brull e colaboradores^(28)^ comparou três metodologias quanto à retenção de conhecimento dos participantes no pós-teste. Foram comparadas aulas expositivas, aulas on-line e um jogo interativo. Todos os grupos, apesar de terem recebido o mesmo conteúdo, apresentaram diferenças nos resultados do pós-teste. O grupo que utilizou o jogo obteve um número maior de respostas corretas em comparação aos outros dois grupos.

O uso de materiais educativos exige um cuidado metodológico para contemplar os objetivos a serem alcançados. A construção do jogo de tabuleiro, utilizando o método proposto por Andrade e colaboradores^(12)^, apresenta as etapas essenciais para a criação de um produto pedagógico. Dessa forma, as atividades lúdicas favorecem habilidades profissionais como: análise crítica, relações sociais, criatividade, imaginação e motivação^(27)^.

A validação de uma tecnologia educacional é necessária para a legitimidade do produto produzido. Todos os itens devem ser representativos e claros, de forma a serem adequadamente utilizados para a função para a qual foram criados. As contribuições incorporadas permitem a concepção científica do produto, respaldando seu alcance educativo^(29)^. A construção e validação do conteúdo do jogo mostraram-se adequadas, com consenso dos juízes sobre os temas apresentados, sendo necessária apenas uma rodada entre os especialistas. A validação de conteúdo representou a integração das atividades da prática da pesquisadora com o referencial teórico científico. Vinte cartas atingiram o consenso recomendado, enquanto seis foram excluídas por não atingirem o IVC necessário. Os juízes expressaram suas opiniões sobre a maioria das cartas, e as adequações sugeridas foram realizadas.

A avaliação realizada pelo público ao qual se destina o produto é importante para verificar a necessidade de adequações, garantindo que o material seja prático e acessível^(30)^. Não ocorre o questionamento do conteúdo, mas sim a exposição das opiniões sobre objetivos, organização, estilo da escrita, aparência e motivação^(15)^. As respostas fornecidas pelos participantes do Grupo B representaram o encerramento do processo de construção do jogo, o que contribuiu para uma resposta positiva à ação educativa.

### Limitações do estudo

O jogo foi construído com base na realidade local, atendendo às necessidades apresentadas pelos participantes.

### Contribuições para a área da enfermagem, saúde ou política pública

Este produto poderá ser utilizado por diversos serviços de saúde em todo o território nacional, com o objetivo de aprimorar o conhecimento dos profissionais de enfermagem em relação às medidas de biossegurança na CME. Salienta-se também a sugestão de estudos futuros que investiguem e comparem o aprendizado utilizando jogos educacionais.

## CONCLUSÕES

A pesquisa atingiu seu objetivo de construir e validar um jogo educativo. O jogo de tabuleiro “Minha Saúde em Primeiro Lugar” demonstrou que atende ao propósito de proporcionar aos profissionais de enfermagem o reconhecimento das fragilidades relacionadas à biossegurança no ambiente de trabalho de uma Central de Material e Esterilização, incentivando-os a adotar medidas de segurança para prevenir o adoecimento e os acidentes relacionados ao trabalho. As discussões nos grupos desta pesquisa contribuíram para as observações necessárias no protótipo, aproximando-o da realidade vivenciada na prática profissional. As preocupações dos participantes estavam refletidas nas cartas do jogo, confirmando os conteúdos selecionados pela pesquisadora durante a construção das cartas. Ao refletir sobre a prática profissional e correlacionar os riscos ocupacionais presentes, os profissionais foram capazes de promover ações seguras, identificando problemas e buscando soluções.

A participação ativa dos profissionais de enfermagem envolvidos na construção do jogo favoreceu a concepção das necessidades reais da prática profissional. Muitas vezes, as ações educativas são desenvolvidas na teoria, sem atender à realidade local. As atividades de capacitação, sejam por educação continuada ou permanente, representam um desafio para os gestores. A adesão dos trabalhadores a essas capacitações é dificultada pelas rotinas impostas pelo serviço, que não proporcionam espaço para a educação permanente em serviço, o que desmotiva os profissionais de saúde a participarem.
